# *rac*-2-*tert*-Butyl-2,4,5,6,6-penta­chloro­cyclo­hex-3-en-1-one

**DOI:** 10.1107/S160053681100897X

**Published:** 2011-03-15

**Authors:** Abel M. Maharramov, Mirza A. Allahverdiyev, Elmar Y. Mammadov, Ayten R. Askerova, Bahruz A. Rashidov

**Affiliations:** a Baku State University, Z. Khalilov St. 23, Baku AZ-1148, Azerbaijan

## Abstract

The title compound, C_10_H_11_Cl_5_O, is a chiral mol­ecule with two stereogenic centres. However, it crystallizes as a racemate. One of enanti­omers reveals the relative configuration (2S*,5R*). The cyclo­hexene ring adopts a half-chair conformation.

## Related literature

For general background to the synthesis of 2-*tert*-butyl-2,4,5,6,6-penta­chloro­cyclo­hex-3-enone and its derivatives, see: Hartshorn *et al.* (1992 ▶).
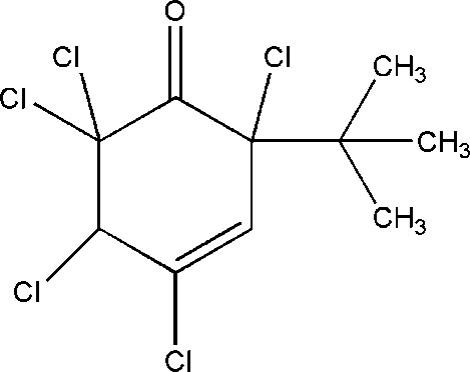


## Experimental

### 

#### Crystal data

C_10_H_11_Cl_5_O
*M* *_r_* = 324.44
Monoclinic, 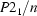

*a* = 6.9466 (7) Å
*b* = 15.7237 (15) Å
*c* = 12.1785 (13) Å
β = 94.903 (5)°
*V* = 1325.3 (2) Å^3^
*Z* = 4
Mo *K*α radiation
μ = 1.07 mm^−1^
*T* = 296 K
0.30 × 0.30 × 0.20 mm

#### Data collection

Bruker APEXII CCD diffractometer
Absorption correction: multi-scan (*SADABS*; Sheldrick, 2003 ▶) *T*_min_ = 0.740, *T*_max_ = 0.815
11583 measured reflections
2880 independent reflections
2472 reflections with *I* > 2σ(*I*)
*R*_int_ = 0.024

#### Refinement

*R*[*F* ^2^ > 2σ(*F* ^2^)] = 0.031
*wR*(*F* ^2^) = 0.082
*S* = 1.00
2880 reflections
148 parameters
H-atom parameters constrained
Δρ_max_ = 0.39 e Å^−3^
Δρ_min_ = −0.23 e Å^−3^

### 

Data collection: *APEX2* (Bruker, 2005 ▶); cell refinement: *SAINT-Plus* (Bruker, 2001 ▶); data reduction: *SAINT-Plus*; program(s) used to solve structure: *SHELXTL* (Sheldrick, 2008 ▶); program(s) used to refine structure: *SHELXTL*; molecular graphics: *SHELXTL*; software used to prepare material for publication: *SHELXTL*.

## Supplementary Material

Crystal structure: contains datablocks global, I. DOI: 10.1107/S160053681100897X/kp2308sup1.cif

Structure factors: contains datablocks I. DOI: 10.1107/S160053681100897X/kp2308Isup2.hkl

Additional supplementary materials:  crystallographic information; 3D view; checkCIF report

## Figures and Tables

**Fig. 1.**
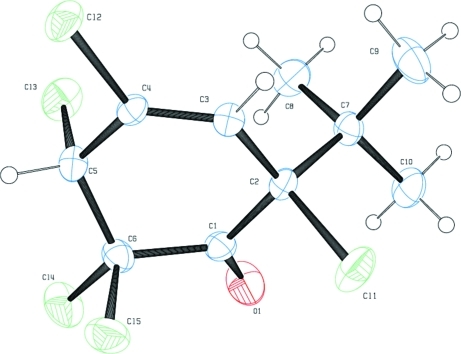
The molecular structure of the title compound with the atomic numbering scheme. Displacement ellipsoids are drawn at the 30% probability level.

## supplementary crystallographic information

### Comment

The synthesis of 2-*tert*-butyl-2,4,5,6,6-pentachlorocyclohex-3-enone and derivatives has been studied (Hartshorn *et al.*, 1992). Here we describe recystallisation and structural characteristics of the target compound. In the title compound, C_10_H_11_Cl_5_O (I), the cyclohexene ring adopts a half-chair conformation (Fig. 1). The tert-butyl group is attached in a pseudo-axial position. Torsion angle between the tert-butyl group and the carbonyl group is O1—C1—C1—C7 is 39.5 (3) °. The molecule (I) possess two stereogenic centres at C2 and C5 carbon atoms. The crystal of (I) is racemate and consists of enantiomeric pairs with the relative configuration 2S*,5*R**. The crystal packing is realised by van der Waals interactions.

### Experimental

2,6-di-tert-butylphenol (2.30 mol) and ammonium thiocyanate (4.83 mol) in methanol (1200 mL) was stirred with cooling at 273 K. While the temperature was kept in the range from 273 K to 283 K, chlorine gas was slowly bubbled through the mixture for about 1 h, during which the reaction mixture turned to yellow colour. Ammonia was then bubbled through the mixture for about 1.5 h, keeping the reaction mixture in the temperature range from 273 K to 283 K. The reaction was stirred for one more h at 273 K, poured into 2 L of cold distilled water and refrigerated overnight. The aqueous phase was decanted and the solid was taken up in methanol, precipitated by addition of water, filtered and dried. The resulting gummy yellow solid was recrystallised from methanol and dried in vacuum to yield the product as a white powder. The crystals were dissolved in methanol and recrystallised to yield colourless block-shaped crystals of the title compound.

### Refinement

The other hydrogen atoms were placed in calculated positions with and refined in the riding model with fixed isotropic displacement parameters [*U*_iso_(H) = 1.2*U*_eq_(C)].

### Crystal data

**Table:**

C_10_H_11_Cl_5_O	*F*(000) = 656
*M**_r_* = 324.44	*D*_x_ = 1.626 Mg m^−^^3^
Monoclinic, *P*2_1_/*n*	Mo *K*α radiation, λ = 0.71073 Å
Hall symbol: -P 2yn	Cell parameters from 6043 reflections
*a* = 6.9466 (7) Å	θ = 2.6–28.3°
*b* = 15.7237 (15) Å	µ = 1.07 mm^−^^1^
*c* = 12.1785 (13) Å	*T* = 296 K
β = 94.903 (5)°	Prism, colourless
*V* = 1325.3 (2) Å^3^	0.30 × 0.30 × 0.20 mm
*Z* = 4	

### Data collection

**Table:**

Bruker APEXII CCD diffractometer	2880 independent reflections
Radiation source: fine-focus sealed tube	2472 reflections with *I* > 2σ(*I*)
graphite	*R*_int_ = 0.024
φ and ω scans	θ_max_ = 27.0°, θ_min_ = 2.1°
Absorption correction: multi-scan (*SADABS*; Sheldrick, 2003)	*h* = −8→8
*T*_min_ = 0.740, *T*_max_ = 0.815	*k* = −19→20
11583 measured reflections	*l* = −13→15

### Refinement

**Table:**

Refinement on *F*^2^	Primary atom site location: structure-invariant direct methods
Least-squares matrix: full	Secondary atom site location: difference Fourier map
*R*[*F*^2^ > 2σ(*F*^2^)] = 0.031	Hydrogen site location: difference Fourier map
*wR*(*F*^2^) = 0.082	H-atom parameters constrained
*S* = 1.00	*w* = 1/[σ^2^(*F*_o_^2^) + (0.0394*P*)^2^ + 0.5385*P*] where *P* = (*F*_o_^2^ + 2*F*_c_^2^)/3
2880 reflections	(Δ/σ)_max_ = 0.001
148 parameters	Δρ_max_ = 0.39 e Å^−^^3^
0 restraints	Δρ_min_ = −0.23 e Å^−^^3^

### Special details

**Table:**

Geometry. All e.s.d.'s (except the e.s.d. in the dihedral angle between two l.s. planes) are estimated using the full covariance matrix. The cell e.s.d.'s are taken into account individually in the estimation of e.s.d.'s in distances, angles and torsion angles; correlations between e.s.d.'s in cell parameters are only used when they are defined by crystal symmetry. An approximate (isotropic) treatment of cell e.s.d.'s is used for estimating e.s.d.'s involving l.s. planes.
Refinement. Refinement of *F*^2^ against ALL reflections. The weighted *R*-factor *wR* and goodness of fit *S* are based on *F*^2^, conventional *R*-factors *R* are based on *F*, with *F* set to zero for negative *F*^2^. The threshold expression of *F*^2^ > σ(*F*^2^) is used only for calculating *R*-factors(gt) *etc*. and is not relevant to the choice of reflections for refinement. *R*-factors based on *F*^2^ are statistically about twice as large as those based on *F*, and *R*- factors based on ALL data will be even larger.

### Fractional atomic coordinates and isotropic or equivalent isotropic displacement parameters (Å^2^)

**Table:**

	*x*	*y*	*z*	*U*_iso_*/*U*_eq_	
Cl1	0.71222 (8)	0.85115 (3)	0.19948 (5)	0.05953 (16)	
Cl2	1.04573 (7)	0.57732 (3)	0.09467 (4)	0.04698 (13)	
Cl3	1.04201 (9)	0.55909 (4)	0.38510 (5)	0.06249 (17)	
Cl4	0.60933 (10)	0.59189 (4)	0.43935 (5)	0.0725 (2)	
Cl5	0.51257 (7)	0.65020 (4)	0.21735 (5)	0.05830 (16)	
O1	0.7230 (3)	0.76496 (11)	0.44023 (14)	0.0731 (5)	
C1	0.7672 (3)	0.73432 (11)	0.35685 (15)	0.0392 (4)	
C2	0.8874 (2)	0.78139 (10)	0.27498 (13)	0.0332 (3)	
C3	0.9594 (2)	0.72330 (10)	0.18964 (13)	0.0322 (3)	
H3	1.0140	0.7479	0.1302	0.039*	
C4	0.9500 (2)	0.63939 (10)	0.19366 (13)	0.0311 (3)	
C5	0.8610 (3)	0.58988 (11)	0.28024 (14)	0.0382 (4)	
H5	0.8030	0.5383	0.2464	0.046*	
C6	0.7021 (3)	0.64222 (12)	0.32611 (15)	0.0412 (4)	
C7	1.0544 (3)	0.83642 (11)	0.33343 (15)	0.0393 (4)	
C8	1.1855 (3)	0.77762 (14)	0.40573 (18)	0.0555 (5)	
H8A	1.1150	0.7548	0.4635	0.083*	
H8B	1.2949	0.8091	0.4374	0.083*	
H8C	1.2290	0.7319	0.3618	0.083*	
C9	1.1720 (4)	0.87761 (16)	0.2469 (2)	0.0649 (6)	
H9A	1.2300	0.8341	0.2054	0.097*	
H9B	1.2713	0.9127	0.2830	0.097*	
H9C	1.0884	0.9119	0.1981	0.097*	
C10	0.9811 (3)	0.90805 (13)	0.40509 (18)	0.0549 (5)	
H10A	1.0887	0.9412	0.4357	0.082*	
H10B	0.9150	0.8838	0.4637	0.082*	
H10C	0.8939	0.9439	0.3606	0.082*	

### Atomic displacement parameters (Å^2^)

**Table:**

	*U*^11^	*U*^22^	*U*^33^	*U*^12^	*U*^13^	*U*^23^
Cl1	0.0644 (3)	0.0425 (3)	0.0680 (3)	0.0196 (2)	−0.0158 (3)	−0.0037 (2)
Cl2	0.0602 (3)	0.0397 (2)	0.0426 (3)	0.0075 (2)	0.0136 (2)	−0.01185 (18)
Cl3	0.0763 (4)	0.0583 (3)	0.0519 (3)	0.0143 (3)	−0.0003 (3)	0.0212 (2)
Cl4	0.0883 (5)	0.0718 (4)	0.0632 (4)	−0.0239 (3)	0.0395 (3)	0.0014 (3)
Cl5	0.0372 (2)	0.0698 (4)	0.0671 (3)	−0.0054 (2)	−0.0002 (2)	−0.0178 (3)
O1	0.0907 (12)	0.0659 (10)	0.0698 (11)	−0.0203 (9)	0.0479 (10)	−0.0304 (8)
C1	0.0370 (9)	0.0393 (9)	0.0423 (10)	0.0002 (7)	0.0098 (7)	−0.0097 (7)
C2	0.0370 (9)	0.0268 (8)	0.0353 (9)	0.0040 (6)	0.0009 (7)	−0.0029 (6)
C3	0.0372 (9)	0.0322 (8)	0.0270 (8)	0.0004 (7)	0.0026 (6)	−0.0002 (6)
C4	0.0335 (8)	0.0311 (8)	0.0286 (8)	0.0034 (6)	0.0025 (6)	−0.0050 (6)
C5	0.0482 (10)	0.0295 (8)	0.0372 (9)	−0.0016 (7)	0.0051 (7)	−0.0005 (7)
C6	0.0421 (10)	0.0421 (10)	0.0409 (10)	−0.0075 (8)	0.0116 (8)	−0.0024 (8)
C7	0.0442 (10)	0.0331 (9)	0.0405 (10)	−0.0044 (7)	0.0024 (8)	−0.0088 (7)
C8	0.0505 (12)	0.0561 (12)	0.0570 (13)	0.0041 (10)	−0.0118 (9)	−0.0138 (10)
C9	0.0754 (16)	0.0562 (13)	0.0650 (14)	−0.0310 (12)	0.0174 (12)	−0.0111 (11)
C10	0.0675 (14)	0.0396 (10)	0.0570 (13)	0.0000 (10)	0.0016 (10)	−0.0186 (9)

### Geometric parameters (Å, °)

**Table:**

Cl1—C2	1.8271 (17)	C5—H5	0.9800
Cl2—C4	1.7278 (16)	C7—C8	1.524 (3)
Cl3—C5	1.7808 (19)	C7—C9	1.531 (3)
Cl4—C6	1.7593 (18)	C7—C10	1.538 (2)
Cl5—C6	1.790 (2)	C8—H8A	0.9600
O1—C1	1.188 (2)	C8—H8B	0.9600
C1—C2	1.543 (2)	C8—H8C	0.9600
C1—C6	1.553 (2)	C9—H9A	0.9600
C2—C3	1.501 (2)	C9—H9B	0.9600
C2—C7	1.569 (2)	C9—H9C	0.9600
C3—C4	1.322 (2)	C10—H10A	0.9600
C3—H3	0.9300	C10—H10B	0.9600
C4—C5	1.487 (2)	C10—H10C	0.9600
C5—C6	1.521 (3)		
			
O1—C1—C2	123.48 (16)	Cl4—C6—Cl5	108.43 (10)
O1—C1—C6	119.52 (17)	C8—C7—C9	109.08 (18)
C2—C1—C6	117.00 (14)	C8—C7—C10	109.08 (16)
C3—C2—C1	112.72 (13)	C9—C7—C10	107.62 (17)
C3—C2—C7	111.88 (14)	C8—C7—C2	108.04 (14)
C1—C2—C7	113.04 (14)	C9—C7—C2	109.78 (15)
C3—C2—Cl1	105.30 (11)	C10—C7—C2	113.17 (16)
C1—C2—Cl1	103.84 (11)	C7—C8—H8A	109.5
C7—C2—Cl1	109.40 (11)	C7—C8—H8B	109.5
C4—C3—C2	124.13 (15)	H8A—C8—H8B	109.5
C4—C3—H3	117.9	C7—C8—H8C	109.5
C2—C3—H3	117.9	H8A—C8—H8C	109.5
C3—C4—C5	125.00 (15)	H8B—C8—H8C	109.5
C3—C4—Cl2	120.98 (13)	C7—C9—H9A	109.5
C5—C4—Cl2	114.03 (12)	C7—C9—H9B	109.5
C4—C5—C6	109.63 (14)	H9A—C9—H9B	109.5
C4—C5—Cl3	109.88 (12)	C7—C9—H9C	109.5
C6—C5—Cl3	111.96 (13)	H9A—C9—H9C	109.5
C4—C5—H5	108.4	H9B—C9—H9C	109.5
C6—C5—H5	108.4	C7—C10—H10A	109.5
Cl3—C5—H5	108.4	C7—C10—H10B	109.5
C5—C6—C1	112.78 (14)	H10A—C10—H10B	109.5
C5—C6—Cl4	111.74 (13)	C7—C10—H10C	109.5
C1—C6—Cl4	110.43 (13)	H10A—C10—H10C	109.5
C5—C6—Cl5	106.09 (12)	H10B—C10—H10C	109.5
C1—C6—Cl5	107.08 (13)		
			
O1—C1—C2—C3	167.6 (2)	Cl3—C5—C6—Cl4	−51.23 (16)
C6—C1—C2—C3	−12.6 (2)	C4—C5—C6—Cl5	68.58 (15)
O1—C1—C2—C7	39.5 (3)	Cl3—C5—C6—Cl5	−169.21 (9)
C6—C1—C2—C7	−140.75 (16)	O1—C1—C6—C5	−137.5 (2)
O1—C1—C2—Cl1	−79.0 (2)	C2—C1—C6—C5	42.7 (2)
C6—C1—C2—Cl1	100.79 (15)	O1—C1—C6—Cl4	−11.7 (2)
C1—C2—C3—C4	−10.6 (2)	C2—C1—C6—Cl4	168.56 (13)
C7—C2—C3—C4	118.08 (18)	O1—C1—C6—Cl5	106.2 (2)
Cl1—C2—C3—C4	−123.17 (16)	C2—C1—C6—Cl5	−73.58 (17)
C2—C3—C4—C5	2.4 (3)	C3—C2—C7—C8	−69.95 (19)
C2—C3—C4—Cl2	−177.26 (12)	C1—C2—C7—C8	58.59 (19)
C3—C4—C5—C6	28.3 (2)	Cl1—C2—C7—C8	173.76 (13)
Cl2—C4—C5—C6	−152.03 (13)	C3—C2—C7—C9	48.9 (2)
C3—C4—C5—Cl3	−95.12 (18)	C1—C2—C7—C9	177.43 (16)
Cl2—C4—C5—Cl3	84.53 (13)	Cl1—C2—C7—C9	−67.40 (18)
C4—C5—C6—C1	−48.3 (2)	C3—C2—C7—C10	169.15 (16)
Cl3—C5—C6—C1	73.88 (17)	C1—C2—C7—C10	−62.3 (2)
C4—C5—C6—Cl4	−173.43 (12)	Cl1—C2—C7—C10	52.87 (18)
